# The Effect of Clozapine and Novel Glutamate Modulator JNJ-46356479 on Nitrosative Stress in a Postnatal Murine Ketamine Model of Schizophrenia

**DOI:** 10.3390/ijms24021022

**Published:** 2023-01-05

**Authors:** Nina Treder, Albert Martínez-Pinteño, Natalia Rodríguez, Néstor Arbelo, Santiago Madero, Marta Gómez, Clemente García-Rizo, Sergi Mas, Patricia Gassó, Eduard Parellada, Constanza Morén

**Affiliations:** 1 Faculty of Psychology and Neuroscience, Maastricht University, 6211 LK Maastricht, The Netherlands; 2 Pharmacology Unit, Department of Basic and Clinical Practice, University of Barcelona, 08036 Barcelona, Spain; 3 Clinical and Experimental Neuroscience Area, The August Pi i Sunyer Biomedical Research Institute (IDIBAPS), 08036 Barcelona, Spain; 4 Barcelona Clínic Schizophrenia Unit, Institute of Neuroscience, Hospital Clínic of Barcelona, 08036 Barcelona, Spain; 5 G04 Group, Centro de Investigación Biomédica en Red de Salud Mental (CIBERSAM), Carlos III Health Institute, 28029 Madrid, Spain; 6 Hospital Álvaro Cunqueiro, Complejo Hospitalario Universitario de Vigo (SERGAS), 36312 Vigo, Spain; 7 Instituto de Investigación Sanitaria Galicia Sur (IISGS), 36312 Vigo, Spain; 8 Centro de Investigación Biomédica en Red de Enfermedades Raras (CIBERER), Carlos III Health Institute, 28029 Madrid, Spain

**Keywords:** clozapine, glutamate metabotropic modulator, ketamine, nitrosative stress, schizophrenia

## Abstract

Schizophrenia (SZ) is a heterogeneous mental disorder, affecting ~1% of the worldwide population. One of the main pathophysiological theories of SZ is the imbalance of excitatory glutamatergic pyramidal neurons and inhibitory GABAergic interneurons, involving N-methyl-D-aspartate receptors (NMDAr). This may lead to local glutamate storms coupled with excessive dendritic pruning and subsequent cellular stress, including nitrosative stress, during a critical period of neurodevelopment, such as adolescence. Nitrosative stress is mediated by nitric oxide (NO), which is released by NO synthases (NOS) and has emerged as a key signaling molecule implicated in SZ. Regarding glutamatergic models of SZ, the administration of NMDAr antagonists has been found to increase NOS levels in the prefrontal cortex (PFC) and ventral hippocampus (HPC). We hypothesized that suboptimal NOS function in adolescence could be a target for early treatments, including clozapine (CLZ) and the novel metabotropic glutamate receptor modulator JNJ-46356479 (JNJ). We analyzed the protein levels of NOS isoforms in adult PFC and HPC of a postnatal ketamine induced murine model of SZ receiving CLZ or JNJ during adolescence by western blot. Endothelial NOS and neuronal NOS increased under ketamine administration in PFC and decreased in CLZ or JNJ treatments. The same trends were found in the HPC in neuronal NOS. In contrast, inducible NOS was increased under JNJ treatment with respect to ketamine induction in the HPC, and the same trends were found in the PFC. Taken together, our findings suggest a misbalance of the NOS system following NMDAr antagonist administration, which was then modulated under early CLZ and JNJ treatments.

## 1. Introduction

Schizophrenia (SZ) is a severe psychiatric disorder with unknown etiopathogenesis that affects approximately 1% of the worldwide population [1]. SZ is characterized by positive (e.g., hallucinations), negative (e.g., apathy, social withdrawal), and cognitive (e.g., deficits of executive functioning and working memory) symptoms [2,3].

SZ is defined as corresponding to a complex phenotype due to gene–environment interactions [4]. According to the polygenic theory of SZ, a large fraction of the genetic risk is explained by many common genetic variants in multiple genes with very small effect sizes. The advancement of genome-wide association studies (GWAS) in large, well-powered samples in psychiatry has allowed the estimation of genetic predisposition using genetic constructs such as polygenic risk scores (PRS). PRS for SZ, bipolar disorder, and cognitive functions are linked to symptom severity and recovery, comorbid conditions, and cognitive functioning [5,6]. Unfortunately, genetic biomarkers explain only a small fraction of the observed variability in treatment response and cannot be included in clinical practice to guide treatment strategies. Currently, treatment options include clozapine (CLZ), which is the first atypical antipsychotic primarily used to treat SZ patients and schizoaffective disorders who have had an inadequate response to other antipsychotics or who have been unable to tolerate other drugs due to extrapyramidal side effects, whereas metabotropic glutamate receptor 2 (mGlu2r) negatively modulates glutamate release and is considered a potential clinical target for novel antipsychotic drugs [7].

The efficacy of most treatment regimens is evaluated during clinical stages, when positive and negative diagnostic features have already surfaced, despite that cognitive impairments emerge years before the disorder and can be clinically diagnosed at the prodromal stage [8]. To date, while positive symptoms can be targeted by classical antipsychotics, negative and cognitive symptoms remain refractory to them [9]. Thus, directing SZ research toward the identification of early biomarkers and novel treatment options during prodromal stages is an urgent need to effectively prevent or halt the onset of SZ.

While past pharmacological research has directed efforts to antagonizing the hyperdopaminergic and hyperserotonergic states underlying positive symptoms [3], novel brain imaging studies converge on supporting the “glutamate hypothesis” that seems to also explain the negative and cognitive symptoms of SZ [9]. This hypothesis mainly imputes the development of SZ symptoms to the reduced signaling of ionotropic NMDA glutamate receptors (NMDAr) and defects in inhibitory parvalbumin positive (PV+) GABA interneurons [10]. Accordingly, increasing evidence points to glutamate excitotoxicity [11,12]. Such neurotoxic events are thought to increase synaptic pruning, leading to loss of dendritic spine density in neurons of the prefrontal cortex (PFC) and hippocampus (HPC) and underpin the development of cognitive symptoms of SZ [13]. In fact, changes in both gray matter volume and white matter integrity in PFC and HPC areas occurring in the onset of SZ [14] are linked to early negative and cognitive symptoms [15].

In line with the abovementioned glutamatergic excitotoxicity, when glutamate binds to NMDAr, NMDAr-gated channels become permeable to Ca^2+^, and this Ca^2+^ flux binds to calmodulin and stimulates the neuronal nitric oxide synthase (NOS) enzyme to produce nitric oxide (NO) in the central nervous system [16]. Subsequent reactions modulate the release of neurotransmitters such as glutamate and dopamine and are critical for the refinement and maintenance of synaptic connections, long-term potentiation, and memory formation [16]. Based on this, increases of reactive nitrogen species or nitrosative stress related to abnormal levels of NO have been associated with neuronal injury in SZ [17,18] and are considered an emerging pathological process in the disease [19] and a potential target for treatment development [20]. In summary, as an important intercellular messenger in the central nervous system, NO is involved in synaptic plasticity [21,22], neurotransmitter release including dopamine and glutamate [23], cell survival [24], and peroxidation and oxidative stress [25], all involved in the pathophysiology of SZ. On one hand, human data show a key involvement of NO in SZ: (i) NO was found to present a potential epigenetic role in SZ by modulating methylation, acetylation, or gene expression among others [26]; (ii) nicotinamide-adenine dinucleotide phosphate-diaphorase (NADPH-d) is an NOS capable of generating NO [27,28], and interestingly, NADPH-d neurons were found to migrate improperly in the PFC, HPC formation, and lateral temporal lobe of SZ patients [29,30]; (iii) postmortem studies described an increase of NOS in SZ patients [31,32,33]. On the other hand, animal data also revealed NO involvement in SZ: (i) in genetic models, neuronal NOS knock-out mice displayed SZ-related behaviors [34]; (ii) in glutamatergic models of SZ, phencyclidine administration increased levels in the PFC and ventral HPC [35]. Taken together, the role of NO was implicated in the coordination of pre- and post-synaptic changes at excitatory synapses, leading to the modulation of synaptic plasticity in both excitatory and inhibitory synapses, particularly in the PFC and HPC [36], and last but not least, the administration of NO donors can reverse SZ-related behavioral deficits [37].

In this study we used a postnatal ketamine (Ket) mouse model of SZ to assess whether nitrosative factors, such as the three different endothelial (eNOS), neuronal (nNOS), and inducible (iNOS) isoforms of NOS [38], lay at the basis of SZ etiopathogenesis and if they are also affected by early treatment approaches. In our rodent model, the administration of the NMDAr antagonist Ket on postnatal days (PNDs) 7, 9, and 11 in mice showed to reproduce negative and cognitive symptoms that persevere throughout adulthood (Jeevakumar et al., 2015; Martínez-Pinteño et al., 2020). Moreover, mice treated with Ket also showed a reduction in PV+ in the mPFC and dentate gyrus, together with an increase in c-Fos expression in this HPC area [7]. Thus, we aimed to explore the effect of early treatments that were administered during adolescence with clozapine CLZ and the novel positive allosteric modulator (PAM) of mGlu2r JNJ-46356479 (JNJ) developed by Janssen-Cilag [39] that inhibits glutamate presynaptic release [39,40,41] on nitrosative factors in adult SZ-like mice.

## 2. Results

### 2.1. Effect of Pharmacological Treatment (CLZ or JNJ) on NOS Isoforms in the Vehicle Groups

The treatment effects on eNOS, nNOS, and iNOS protein levels in the control groups treated with the vehicle are shown in Supplementary Table S2. No significant effects associated with treatment were found in the vehicle group, except for an increase in eNOS and nNOS after CLZ administration and a decrease in eNOS after JNJ administration in the PFC.

### 2.2. Effect of Pharmacological Treatment (CLZ or JNJ) on NOS Isoforms in the PFC of the SZ-Like Groups Compared to Controls

**Figure 1:**
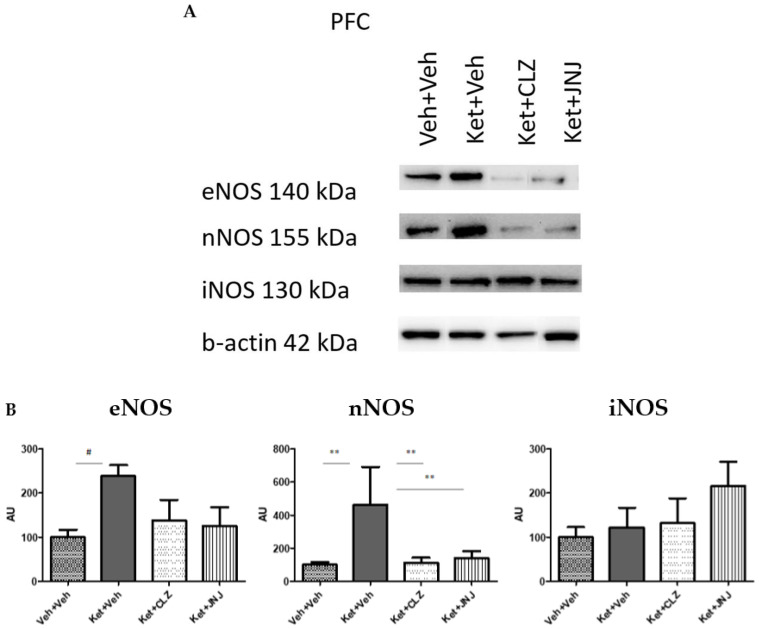
Effect of CLZ and JNJ treatments on protein levels of NOS isoforms in the PFC of SZ-like mice induced with Ket. (**A**) Representative densitometry blots of eNOS, nNOS, and iNOS isoforms in the PFC tissue from the different groups of study, including vehicle controls, SZ-like mice induced with Ket, and Ket-induced mice treated with CLZ or JNJ. The upper panels show the three protein isoforms of interest, and the lower panel represents the corresponding housekeeping β-Actin. (**B**) Protein levels of the three NOS isoforms (eNOS, nNOS, and iNOS) were estimated by quantification of densitometry blots (AU). Both eNOS and nNOS increased in the Ket-induced group compared to the vehicle and decreased in the CLZ and JNJ treated groups, although the decrease associated with the pharmacological treatments was only significant in the neuronal isoform. The iNOS isoform did not lead to significant differences among the groups of study. # *p* = 0.07, ** *p* < 0.01 (AU, arbitrary units; CLZ, clozapine; eNOS, endothelial nitric oxide synthase; iNOS, inducible nitric oxide synthase; JNJ, JNJ-46356479; Ket, ketamine; NOS, nitric oxide synthase; nNOS, neuronal nitric oxide synthase; PFC, prefrontal cortex; Veh, vehicle).

The Ket-induced SZ-like group showed increased levels of eNOS and nNOS with respect to the vehicle group ((238.63 ± 24.67 vs. 100 ± 17.41, *p* = 0.07) and (461.80 ± 230.32 vs. 100 ± 15.49, *p* = 0.003), respectively). CLZ and JNJ treatments lowered eNOS and nNOS protein levels when compared to the Ket-induced group, leading to similar levels of the controls ((137.46 ± 47.14, 125.59 ± 42.53, *p* = NS) and (112.25 ± 31.42, 140.25 ± 43.51, *p* = 0.005), respectively), although this decrease was significant only in the neuronal isoform. The protein levels of the iNOS isoform showed a different pattern and did not lead to statistical significances between the different groups of study (Figure 1).

### 2.3. Effect of Pharmacological Treatment (CLZ or JNJ) on NOS Isoforms in the HPC of the SZ-Like Groups Compared to Controls

**Figure 2:**
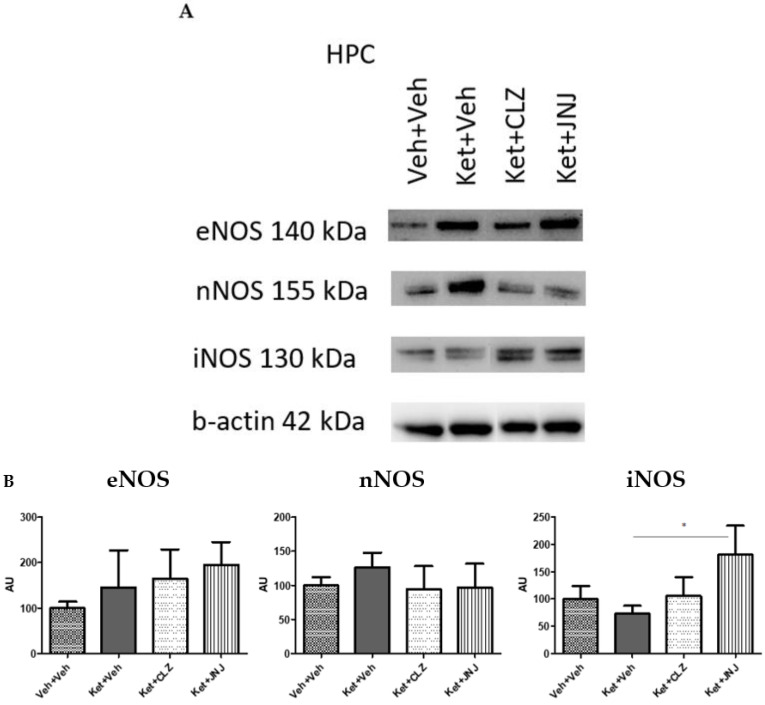
Effect of CLZ and JNJ treatments on protein levels of NOS isoforms in the HPC of SZ-like mice induced with Ket. (**A**) Representative densitometry blots of eNOS, nNOS, and iNOS isoforms in the HPC tissue from the different groups of study, including vehicle controls, SZ-like mice induced with Ket, and Ket-induced mice treated with CLZ or JNJ. The upper panels show the three protein isoforms of interest, and the lower panel represents the corresponding housekeeping β-Actin. (**B**) Protein levels of the three NOS isoforms (eNOS, nNOS, and iNOS) were estimated by quantification of densitometry blots (AU). No significant differences were obtained in the protein levels of eNOS and nNOS isoforms. Protein levels of the inducible iNOS isoform significantly increased in the Ket-induced group treated with JNJ compared to the Ket-induced SZ-like group. * *p* < 0.05 (AU, arbitrary units; CLZ, clozapine; eNOS, endothelial nitric oxide synthase; HPC, hippocampus; iNOS, inducible nitric oxide synthase; JNJ, JNJ-46356479; Ket, ketamine; NOS, nitric oxide synthase; nNOS, neuronal nitric oxide synthase; Veh, vehicle).

No significant differences were found in eNOS and nNOS isoforms in the HPC from the different groups of study. Contrarily, the protein levels of the inducible iNOS isoform significantly increased in the Ket-induced mice treated with JNJ with respect to the Ket-induced SZ-like mice (181.37 ± 53.23 vs. 72.78 ± 15.26, *p* = 0.039) (Figure 2).

Where trends of eNOS protein levels between groups in the HPC differed from the findings in PFC, nNOS levels in both PFC and HPC tissues showed similar patterns (with higher protein levels in the Ket-induced SZ-like mice compared to the vehicle group that were restored under CLZ or JNJ treatments). These findings suggest that Ket exposure leads to an increase in eNOS and nNOS levels both in tissues and CLZ and JNJ pharmacological treatments promote lower levels similar to that of controls in nNOS. Contrarily, iNOS isoform levels responded in a different manner, which was similar in both PFC and HPC tissues (with an increase of protein levels in the JNJ-treated Ket-induced group compared to the Ket-induced SZ-like mice).

## 3. Discussion

**Figure 3:**
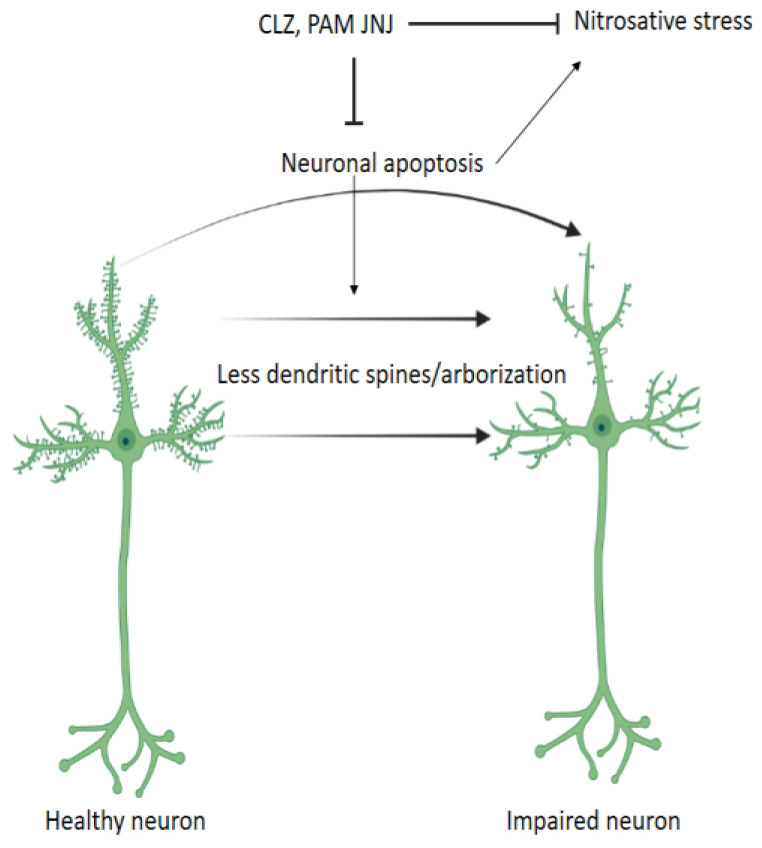
Antipsychotic compounds CLZ and mGlu2r PAM JNJ, among others, may halt both neuronal local apoptosis related to dendritic spines and arborization and subsequent nitrosative stress. CLZ, clozapine; PAM, positive allosteric modulator, JNJ, JNJ-46356479.

The present study was conducted on a postnatal Ket murine model of SZ [7,42] to explore the effect of early treatments, including CLZ and the novel JNJ, on nitrosative factors in PFC and HPC in adult rodents. The administration of the NMDAr antagonist Ket on PND 7, 9, and 11 in mice has been shown to reproduce positive, negative, as well as cognitive symptoms that persevere throughout adulthood [7,42]. To our knowledge, this is the first study investigating the effects of CLZ and novel mGlu2r modulator JNJ on nitrosative factors, when administered during the adolescence period corresponding to PND 35–60, in a postnatal Ket induced mice model of SZ. Such pharmacological therapeutic approaches applied during adolescence may offer great advantages compared to most translational studies investigating antipsychotics, in which pharmacological treatment is usually limited to advanced stages. Due to the neurodevelopmental and neuroprogressive nature of the disease and synaptic over-pruning described during adolescence [1], it is a priority to investigate the effects of early treatment approaches, such as the ones herein conducted. In this study, we analyzed nitrosative stress in PFC and HPC through the assessment of the protein levels of the three known NOS isoforms, eNOS, nNOS, and iNOS, depicted in the literature [38] (Figure 3).

Despite that the etiology of SZ is poorly understood, one of the theories points to disturbances involving NO signaling [35]. NO production in the brain has been associated with both neuroprotective and neurotoxic effects [24], although it was described that NO-derived neurotoxicity occurs especially when overproduced [26]. In fact, excess of NO has been shown to partake in glutamate neurotoxicity in primary neuronal cell culture and animal stroke models [26]. Such evidence supports the feasibility of the glutamatergic model of study with the NMDAr antagonist Ket conducted in this study, and it is aligned with the increased levels of eNOS and nNOS isoforms in the PFC of our SZ-like mice induced with Ket. In fact, glutamatergic models of SZ using phencyclidine administration (other NMDAr antagonists similar to the Ket used in our model) have been shown to increase NOS levels in the PFC and ventral HPC [35], according to our findings of increased eNOS and nNOS levels following Ket induction. In addition, an increase in NO levels, particularly in the PFC and HPC, was observed not only in animal models of SZ but also in the postmortem brains of SZ patients [35].

Based on the validation of our model, we aimed not only to elucidate the potential role of NOS in SZ etiopathogenesis but also to investigate the potential early therapeutic effects of the atypical antipsychotic CLZ and the novel mGlu2r PAM JNJ.

Despite that the atypical antipsychotic CLZ could not inhibit NO release in vitro in microglial cells [43], it was shown to increase social interaction in SZ-like rats treated with the NOS inhibitor [44], demonstrating an influence of CLZ administration in the NOS system. This is in line with our findings of significant reductions of eNOS and nNOS protein levels following CLZ administration in the PFC and non-significant trends in nNOS levels of the HPC. Interestingly, the protein levels of these NOS isoforms were diminished to similar levels of the controls, suggesting potential therapeutic effectiveness in these parameters.

The mGlu2r PAM works by inhibiting the presynaptic release of glutamate [40] and might therefore have the effect of down-regulating the neurotoxic storm of glutamate in the prodromal stages of the disease. This is in line with our findings derived from eNOS and nNOS isoforms in the PFC, in which pharmacological treatment with JNJ led to restored/decreased protein levels similar to the controls, as well as with the trends observed in the nNOS of HPC. Comparable data were obtained in the literature. The administration of the PAM of the mGlu_5_ receptor CDPPB in the olfactory bulbectomized rat model, a well-established model of depression and Alzheimer’s disease, ameliorated cognitive impairment and partially reversed the changes in eNOS and nNOS brain expression induced by the lesion [45]. There are no studies assessing the iNOS isoform response to the early administration of PAM mGlu2r in the literature. In our hands, the treatment effects produced in iNOS followed a different pattern with respect to their endothelial and neuronal counterparts. Although nonsignificant differences were found in the PFC in the different groups of study, a trend towards an increase of this isoform in the Ket + JNJ-treated group was found when compared to the Ket-induced group, which turned out to be significant in the HPC. Additionally, compared to JNJ, CLZ administration did not lead to any changes in iNOS. Altogether, these findings may indicate the involvement of different biological pathways derived from iNOS, with respect to both eNOS and nNOS. Indeed, iNOS features differ from the endothelial and neuronal isoforms. While the first is an inducible, calcium-independent isoform and is mostly involved in inflammatory processes, the latter are constitutive, calcium-dependent isoforms [46], mainly involved in vasodilatation, among other great number of functions [47]. Having this in mind, it is conceivable that the findings derived from iNOS in our study do not follow the same profile observed in both nNOS and eNOS isoforms. In fact, multiple factors may influence NOS responses. For instance, it has been reported that the modulation of NO levels is time-specific, inducing opposite effects depending on the time of administration, i.e., early postnatal age or adulthood [35].

Since our findings were slightly different in PFC and HPC tissues, tissue-specific processes might be at hand. Despite that HPC and PFC have different roles in cognitive functioning, they are both closely linked to SZ [48]; thus, herein, we explored both PFC and HPC tissues as the main target tissues of the disease in order to investigate both SZ etiopathogenesis and pharmacologic approaches. Nevertheless, regional specificity in SZ was already reported before across studies [49] in line with our differential findings in both tissues. However, limitations to this investigation should not be ruled out, such as the sample size and the use of semiquantitative approaches, which may have hampered the level of significance in some cases.

A major issue imposed upon SZ research is the high heterogeneity of the disease and related high rate of comorbidities, which make the disease difficult to model. Therefore, the identification of biomarkers as well as early therapeutic strategies in the prodromal stages is essential. These findings represent a contribution to our understanding of molecular basis in a validated model of SZ, which enables prodromal therapeutic interventions and points out the NOS system as a potential target for the disease.

## 4. Materials and Methods

**Figure 4:**
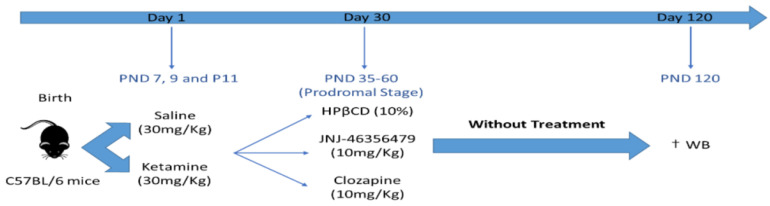
Design of the study. On PND 7, 9, and 11, the C57BL/6J mice were treated with either saline or 30 mg/kg ketamine. This is a study conducted in the prodromal stage of the disease, from 35 to 60 PND, when HPβCD, CLZ, or JNJ were administered daily. On PND 120, mice were sacrificed, and tissues were obtained for further analysis. HPβCD, hydroxypropyl-beta-cyclodextrin; JNJ, JNJ46356479; PND, postnatal days; WB, western blot. † day of sacrification.

An overview of the experimental design is shown in Figure 4.

### 4.1. Animals

For this study, a total of 37 (18 male and 19 female) C57BL/6J mice (Charles River Laboratories, Wilmington, MA, USA) were used. All experimental procedures involving animals were performed in accordance with European Union guidelines on the care and use of laboratory animals and were approved by the University of Barcelona Animal Care Committee and by the Department of the Environment of the Generalitat de Catalunya (Catalonia, Spain) (Code: 386/18 P1, Order Number: 10410, approved on 16 July 2019). Mice were housed in the laboratory animal center of the Faculty of Medicine at the University of Barcelona. After an acclimatization period of 10 days, animals were put in pairs for breeding. Following weaning, mice were subjected to social housing with ad libitum food and drink access at 22 °C in an alternating 12-h light-dark cycle.

### 4.2. Pharmacological Intervention

#### 4.2.1. Postnatal Ketamine Mouse Model

Half of the sex-matched animals (n = 18) were subjected to subcutaneous injections (≈0.1 mL, 25 g needle) of ketamine (30 mg/kg) (Ketamidor 100 mg/mL, Karizoo, Barcelona, Spain) on PNDs 7, 9, and 11 [50], following randomization, to produce a pharmacological model of SZ. The other half sex-matched animals (n = 19) served as a control group and were treated with subcutaneous injections (≈0.1 mL, 25 g needle) of a saline vehicle (NaCl 0.9%, vehicle 1).

#### 4.2.2. Pharmacological Treatment (during Adolescent Period)

The overview of treatment groups and study design is available (Supplementary Table S1). From PND 35 on, animals were randomly allocated to one of three treatment groups. They were treated subcutaneously (≈0.25 mL, 25 g needle) with either vehicle (10% hydroxypropyl-β-cyclodextrin, 5% dimethyl sulfoxide, DMSO, vehicle 2), CLZ (10 mg/kg), or JNJ (10 mg/kg) (Janssen Research and Development, Toledo, Spain) [39] daily until PND 60.

### 4.3. Molecular Tissue Analyses (in Adulthood)

Animals were sacrificed by cervical dislocation on PND 120. Brains were removed, and PFC and HPC were dissected bilaterally. Tissue samples were frozen in liquid nitrogen and stored at −80 °C. PFC and HPC tissue samples were used for the assessment of the three NOS isoforms, eNOS, nNOS, and iNOS [51].

#### 4.3.1. Tissue Lysates

Cell lysis buffer 1× (Cell Signaling Technology, Danvers, MA, USA) with 1 mM of phenylmethylsulfonyl fluoride (PMSF) (Thermo Fisher Scientific, Rockford, IL, USA) was used for the total protein isolation.

#### 4.3.2. Western Blot Analyses

The total protein concentration was assessed using a commercial Lowry assay kit (Bio-rad, Hercules, CA, USA). The homogenized tissue lysates (20 μg total protein) were separated on 4–12% NuPAGE Bis-Tris gels (Invitrogen, Carlsbad, CA, USA) using a MOPS buffer system and were transferred to PVDF membranes (Invitrogen, Carlsbad, CA, USA) using the iBlot 2 Dry Blotting System. The membranes were blocked with 5% nonfat dry milk and TBS-Tween (0.1%) for 1 h. Membranes were probed shaking overnight at 4 °C with the primary antibodies: Mouse anti-NOS1 (1:1000, Santa Cruz Biotechnology, #sc5302, Heidelberg, Germany), Mouse anti-NOS2 (1:1000, Santa Cruz Biotechnology, #sc7271, Heidelberg, Germany), Mouse anti-NOS3 (1:1000, Santa Cruz Biotechnology, #sc376751, Heidelberg, Germany ), Mouse anti-β-actin (1:5000, Sigma Aldrich, #A5441, St Louis, MO, USA). All primary antibodies were prepared in 5% non-fat dry milk (NFDM) and TBS-tween (0.1%) or 5% bovine serum albumin (BSA) in TBS-tween (0.1%). Membranes were then incubated by shaking for 1 h at room temperature with horseradish-peroxidase-conjugated secondary antibodies (Goat Anti-Mouse 1:10,000 to 1:20,000 or Goat anti-Rabbit 1:5000 to 1:10,000) (Thermo Fisher Scientific, Waltham, MA, USA). The membranes were visualized in the Chemidoc system (Bio-rad, Hercules, CA, USA) using the Clarity Western enhanced chemiluminescence (ECL) detection kit (Biorad, Hercules, CA, USA). Protein levels were quantified by measuring the individual protein band intensity from the scanned film by densitometric analysis using Image J version 1.52q (NIH, Bethesda, MD, USA) (Schneider et al., 2012). Protein expression levels were relativized by the housekeeping β-actin as a loading control. Results were expressed as arbitrary units of densitometry.

### 4.4. Statistical Analyses

Statistical analyses were performed with SPSS (version 27.0.1.0, IBM Corp, Chicago, IL, USA). The significance level was set at *p* < 0.05. Data points below or above 1.5× the interquartile range (IQR) were regarded as outliers and excluded from the experimental data prior to statistical analyses. Normality data were confirmed through the Shapiro-Wilk test. Differences between groups were analyzed using a one-way ANOVA, and the mean values for each experimental group were compared using DMS.

## Data Availability

The data presented in this study are available in Supplementary Materials (Tables S1 and S2 and uncropped blots).

## Supplementary Materials

The supporting information can be downloaded at: https://www.mdpi.com/article/10.3390/ijms24021022/s1.
