# Supplementary material for: The Effect of Clozapine and Novel Glutamate Modulator JNJ-46356479 on Nitrosative Stress in a Postnatal Murine Ketamine Model of Schizophrenia
Source: Int J Mol Sci. 2023 Jan 5;24(2):1022. doi: 10.3390/ijms24021022 (PMC9866372; doi:10.3390/ijms24021022)
Supplement: Supplementary file 1 [file ijms-24-01022-s001.zip › 1. Supplementary Table S1.pdf]

Supplementary Table S1.

Overview of the study design and treatment groups.

Group	N	Treatment conditions	PND 7, 9 and 11	PND 35-60	Identifier
Group 1	6	Control	Vehicle 1	Vehicle 2	Veh+Veh
Group 2	7	Control + Clozapine	Vehicle 1	Clozapine (10mg/kg)	Veh+CLZ
Group 3	6	Control + JNJ-46356479	Vehicle 1	JNJ-46356479 (10mg/kg)	Veh+JNJ
Group 4	6	Ketamine	Ketamine (30mg/kg)	Vehicle 2	Ket+Veh
Group 5	6	Ketamine + Clozapine	Ketamine (30mg/kg)	Clozapine (10mg/kg)	Ket+CLZ
Group 6	6	Ketamine + JNJ-46356479	Ketamine (30mg/kg)	JNJ-46356479 (10mg/kg)	Ket+JNJ
**Total**	*37*				

On postnatal days (PNDs) 7, 9 and 11 the animals were treated with either saline or ketamine. On PND 35 to 60 both saline and ketamine mice received either vehicle, clozapine or JNJ-46356479. (CLZ, clozapine; JNJ, JNJ46356479; Ket, ketamine; Veh, vehicle)
