# Supplementary material for: The Effect of Clozapine and Novel Glutamate Modulator JNJ-46356479 on Nitrosative Stress in a Postnatal Murine Ketamine Model of Schizophrenia
Source: Int J Mol Sci. 2023 Jan 5;24(2):1022. doi: 10.3390/ijms24021022 (PMC9866372; doi:10.3390/ijms24021022)
Supplement: Supplementary file 1 [file ijms-24-01022-s001.zip › 2. Supplementary Table S2.pdf]

Supplementary Table S2. Densitometry arbitrary units of protein levels of eNOS, nNOS and iNOS isoforms in PFC and HPC.

	eNOS			nNOS			iNOS		
**PFC**	Average	SEM	p	Average	SEM	p	Average	SEM	p
Veh+veh	100	17.41	-	100	15.49	-	100	22.77	-
Veh+CLZ	351.78	145.35	*	438.22	246.23	*	235.17	84.80	-
Veh+JNJ	66.86	17.96	*	112.20	52.09	-	383.05	189.59	-
	**eNOS**			**nNOS**			**iNOS**		
	Average	SEM	p	Average	SEM	p	Average	SEM	p
**HPC**									
Veh+veh	100	13.77	-	100	12.58	-	100	23.91	-
Veh+CLZ	148.00	68.07	-	118.08	16.48	-	99.87	14.50	-
Veh+JNJ	370.29	214.79	-	194.65	63.04	-	189.54	75.76	-

eNOS, endothelial nitric oxide synthase; HPC, hippocampus; iNOS, inducible nitric oxide synthase; nNOS, neuronal nitric oxide synthase; PFC, prefrontal cortex; SEM, standard error mean; * $p<0.05$.
